# A *taste* of cell-cultured meat: a scoping review

**DOI:** 10.3389/fnut.2024.1332765

**Published:** 2024-01-23

**Authors:** K. V. To, C. C. Comer, S. F. O’Keefe, J. Lahne

**Affiliations:** ^1^Department of Food Science and Technology, Virginia Polytechnic Institute and State University, Blacksburg, VA, United States; ^2^University Libraries, Virginia Polytechnic Institute and State University, Blacksburg, VA, United States

**Keywords:** cultivated meat, sensory evaluation, consumer acceptance, meat alternative, scoping review

## Abstract

Cell-cultured meat (CM) is a novel meat product grown *in vitro* from animal cells, widely framed as equivalent to conventional meat but presented as produced in a more sustainable way. Despite its limited availability for human consumption, consumer acceptance of CM (e.g., willingness to purchase and consume) has been extensively investigated. A key but under-investigated assumption of these studies is that CM’s sensory qualities are comparable to conventional, equivalent meat products. Therefore, the current review aims to clarify what is actually known about the sensory characteristics of CM and their potential impact on consumer acceptance. To this end, a structured scoping review of existing, peer-reviewed literature on the sensory evaluation of CM was conducted according to the PRISMA-ScR and Joanna Briggs Institute guidelines. Among the included studies (*N* = 26), only 5 conducted research activities that could be termed “sensory evaluation,” with only 4 of those 5 studies evaluating actual CM products in some form. The remaining 21 studies based their conclusions on the sensory characteristics of CM and consequent consumer acceptance to a set of hypothetical CM products and consumption experiences, often with explicitly positive information framing. In addition, many consumer acceptance studies in the literature have the explicit goal to increase the acceptance of CM, with some authors (researchers) acting as direct CM industry affiliates; this may be a source of bias on the level of consumer acceptance toward these products. By separating what is known about CM sensory characteristics and consumer acceptance from what is merely speculated, the current review reported realistic expectations of CM’s sensory characteristics within the promissory narratives of CM proponents.

## 1 Introduction

Cell-cultured meat (CM), also known as “lab-grown,” “*in-vitro*,” “cultured meat,” “cultivated meat,” “clean,” “synthetic,” “artificial,” and “cell-based” meat is a meat alternative grown *in vitro* from animal cells using tissue engineering techniques (1, 2). The concept of growing cells *in vitro* was first introduced in 1912 (3), but began to be intensively developed to be used in meat production in the early 2000s (1, 4–6). CM development has been justified for various reasons, from creating a novel food-source for long-term outer space expeditions to ensuring food security in the developing world. Of these, ensuring food sustainability and reducing the environmental impact of current agricultural practices are the two most frequently proposed reasons for developing CM (7–9).

As of mid-2023, over 100 companies worldwide have been involved in the development of CM, both in the meat production from cell lines as well as in lowering the cost of essential components (e.g., growth media or fermentor design) of this technology (10). However, the presence of CM in the market is still very limited. Recently, food agencies in the United States (USDA and FDA) have released regulations regarding human foods made using animal cell culture, and some CM companies (e.g., Upside Foods and Good Meat Inc.) have gained permission to market their products. So far, CM sales have only been approved in the United States and Singapore (11, 12).

Cultured meat has captured the public and scientific imagination across multiple fields. Beyond the technical and scientific challenges to developing such a novel biotechnology, perspectives, challenges, and developments on the topic have frequently been reviewed across many disciplines, from its environmental impacts to the current and possible consumer response to this kind of product (6, 10, 13). Consumer acceptance is a key determinant for the success of any novel food product (14, 15). Therefore, despite its still-extremely limited presence in the marketplace, many studies have been conducted to examine different determinants of consumer acceptance of CM worldwide (15–17). Attitudes toward the biotechnology itself have been studied through survey methodology, as well as the impact of other factors such as consumer demographics (e.g., age, gender, and nationality), psychological characteristics of the consumers (e.g., food neophobia or disgust), and information framing and promissory characteristics around the product [e.g., “Cultured meat is the only alternative to regular meat that consists of real meat. It therefore has the same taste, odor, tenderness, juiciness and mouthfeel as regular meat”; (18), p. 4]; these factors have been evaluated as potential determinants of consumer acceptance (6, 16, 18, 19). Among the tested factors, ‘having similar sensory characteristics as conventional meat’ consistently emerged as a determinant of stated consumer acceptance (20–22).

Meat flavor and texture (tenderness) are the most important sensory characteristics that determine consumer acceptability and purchasing decision of meat products overall (23–25). The limited room for compromise on meat palatability and eating experiences may be a key barrier for consumer adoption of meat substitutes (22, 26, 27). This challenge is especially relevant for CM as it is typically positioned as having the same sensory qualities and functionalities of conventional meat because it is chemically and, in some sense, biologically equivalent (18, 28–30). Because it is arguably “real meat,” it is extremely important that the sensory characteristics of CM align with those expected by consumers for typical meat products. However, due to the extremely limited availability of CM, knowledge of CM sensory characteristics is largely hypothetical, based on CM researchers’ and proponents’ own reports and consumers’ imaginations (28, 31–33).

As the body of literature around CM has grown rapidly, a number of reviews have been published within the broad topic of consumer acceptance of CM (34–36). However, none of these reviews have focused on this topic from the perspective of sensory science to answer critical questions: what are the observable or measurable sensory characteristics of CM, and how are those related to consumer acceptance of CM? Therefore, a scoping review was chosen as a tool to explore the current state of knowledge regarding the sensory characteristics of CM. Scoping reviews are used to map the key concepts and available evidence underpinning a research area (37). This study aimed to identify what is directly known about how CM tastes, how CM is *expected* to taste, and how consumers respond to those sensory characteristics in imagination or reality.

## 2 Materials and methods

### 2.1 Protocol, main question, and definition

This scoping review aimed to answer the question “What are the known sensory characteristics of CM and how have those characteristics been evaluated?” The Preferred Reporting Items for Systematic Reviews and Meta-Analyses guideline for scoping review (PRISMA-ScR) was used as a reporting guideline with the Joanna Briggs Institute (JBI) guidance as the methodological guideline (38). The inclusion and exclusion criteria were defined with an emphasis on the presence of comments on the sensory characteristics of CM regardless of actual product evaluation. CM was defined as a meat alternative produced through animal cell culture to grow meat *in vitro*.

### 2.2 Eligibility criteria

The literature included in this study are peer-reviewed journal articles published between the years 2000 and (June) 2023. The date range was chosen because, even though the concept of CM was introduced in 1912 (4, 5), the approach was not used to produce meat until the early 2000s. To be included in this review, articles had to meet the following criteria:

1. Must be a scholarly (peer-reviewed) publication.
2. The concept or product of cell-cultured meat (defined as “a meat alternative grown *in vitro* from animal cells”) is involved in the study, exclusively or compared to other meat alternatives.
3. Investigates or describes at least one *sensory* characteristic of cell-cultured meat. For this review, we defined sensory characteristics to be both analytic and affective: descriptions of taste or flavors as well as affective responses.
4. Article is written in English.
5. Articles published between January 1, 2000 and June 11, 2023.

Studies with the following characteristics were excluded:

1. Review or non-original research articles. Although not included, citation chasing was conducted on retrieved review articles to ensure maximum scope.
2. Articles focused only on the technical production of cell-cultured meat without evaluation by human subjects.
3. Studies only investigating other meat alternatives (e.g., plants, insects, fungi, etc.).
4. Articles not written in English.
5. Articles published before January 1, 2000.

### 2.3 Information sources and search strategy

Articles from peer-reviewed publications were the primary studies used for this scoping review. The primary studies were retrieved through electronic searches of the following databases: Web of Science Core Collection (WOSCC; Web of Science), Food Science and Technology Abstracts (FSTA; EBSCOhost), and Center for Agriculture and Bioscience International (CABI; CAB Abstracts).

With support and feedback from the University Libraries at Virginia Tech (author CC), the following search strategy was developed iteratively to ensure optimal search results. The final search string as applied in Web of Science is shown in Table 1.

**Table 1:** Search string to identify literature related to cell-cultured meat sensory characteristics in Web of Science, January 20, 2023.

1	(“culture? meat” OR “cultivate? meat” OR “lab-grown meat” OR “cell-based” OR “clean meat” OR “*in-vitro* meat” OR “artificial meat” OR “synthetic meat” OR “cell* meat”)
2	(accept* OR attitude OR preference OR perception OR fram* OR willingness OR awareness OR liking)
3	(taste OR texture OR flavo?r OR appearance OR look OR sensory)
4	#1 AND #2 AND #3

?: To retrieve words with the replacement of 1 character; *: To retrieve words with variant zero to many characters.

Title, abstract, and keywords search was conducted for all concepts in WOSCC and FSTA; all fields were searched in CABI. In all databases, limits were applied using built-in filters for document type (journal article) and publication year (Appendix 1). The format of this search string was modified for application on the other databases. Appendix 1 contains the detailed search strings used in each database. The initial search was performed in January 2023. Considering the rapid increase in publications on CM, monthly searches were performed in each database until June 2023 - the original search strings were reapplied to each database, but limited to (using built-in filters) from 2022 to 2023 to reduce the number of duplicate results. The work cited by articles that were related to topic but did not fully meet criteria (and were excluded) were scanned for additional relevant records (backward citation chasing).

### 2.4 Record screening and data extraction

**Figure 1:**
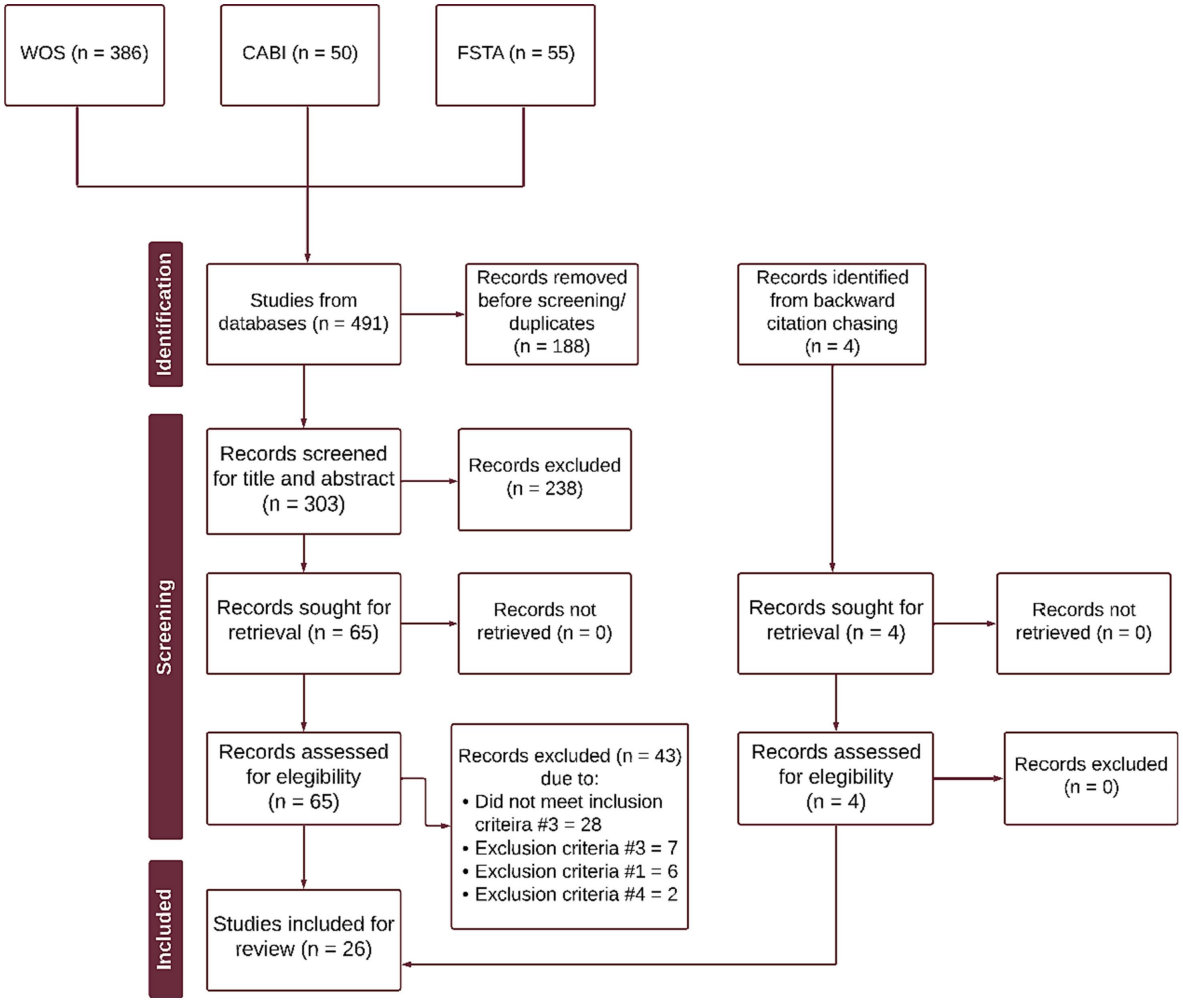
PRISMA flow diagram of the scoping review process. Inclusion criteria #3: Investigates or describes at least one sensory characteristic of cell-cultured meat. For this review, we defined sensory characteristics to be both analytic and affective: descriptions of taste or flavors as well as affective responses. Exclusion criteria #3: Studies only investigating other meat alternatives (e.g., plants, insects, fungi, etc.). Exclusion criteria #1: Review or non-original research articles. Although not included, citation chasing was conducted on retrieved review articles to ensure maximum scope. Exclusion criteria #4: Articles not written in English.

All search results were exported from each database and imported into the systematic review management software Covidence (39), where duplicates identified by the software were removed automatically. Screening proceeded in stages detailed in Figure 1. First, the studies were screened based on the title and abstract, and obviously irrelevant studies were excluded. The full-text of potentially relevant studies was collected and further examined to ensure the studies met eligibility criteria (see Section 2.3). Both title and abstract screening and full-text screening were conducted by two independent reviewers (KT and JL). Discrepancies were resolved through discussions.

Data extraction was conducted in Covidence using a data extraction template developed by the authors. The full data extraction form is attached as Appendix 2. Data extracted included general information (e.g., article title, authors’ name and affiliations, publication year, and DOI), country in which the study is conducted, framed value of CM, sampling methods, survey/sensory evaluation methods, types of sample tested, sensory/acceptance measures, and proposed determinant factors toward CM acceptance, among others. The extraction was focused to identify information related to the expected or actual sensory characteristics of CM, the evidence/ experiment supporting the claim, and the determining factor(s) of CM acceptance. Relevant data were extracted by one author (KT) and reviewed for consistency by a second author (JL).

## 3 Results

The results encompass descriptive characteristics about each study, author-hypothesized determinants of CM acceptance among consumers, and consumer-reported sensory experiences of CM. Consumers’ perceptions of CM’s sensory experiences were treated according to the study method and whether or not they evaluated actual CM samples.

### 3.1 Description of sources

A total of 491 citations were obtained from the database search (WOS = 386 articles, CABI = 50 articles, FSTA = 55 articles) and 4 articles from backward citation chasing. Once duplicates were removed, 303 articles were screened by title and abstract. This first screening excluded 238 publications that were found to be irrelevant to the review objectives. Full texts of the remaining publications were then evaluated according to the eligibility criteria; 43 publications were excluded. Finally, 22 publications from the database search and 4 publications from backward citation chasing were included in the review, resulting in a final count of 26 publications (Figure 1).

Information about each study’s characteristics is presented in Table 2. Of the studies included in this review, most involved participants from the “Global North”: Europe (*N* = 21 studies) and North America (*N* = 7 studies). A minority involved participants from Asian countries (*N* = 5 studies), Australia (*N* = 1 study), and New Zealand (*N* = 1 study). No studies involved participants from countries in South America or Africa. Typically, subjects were selected using simple, random sampling (*N* = 15 studies) or convenience sampling (*N* = 7 studies). Most studies employed online surveys (*N* = 21 studies) while only 2 studies used in-person, paper questionnaires. Qualitative methods (interviewing and focus groups) were in the minority of the selected studies (*N* = 3 studies) and only 5 studies conducted an actual sensory evaluation—using human subjects to assess some aspect of the sensory properties of a food product—to evaluate CM products.

**Table 2:** Characteristics of the included studies based on geographic location, participants selection method, survey method, product (cell-cultured meat) acceptance measures, and if sensory evaluation of cell-cultured meat was considered the main outcome of the study.

Country of studies		Number of studies^a^	References
Belgium	Europe	2	(40, 41)
Croatia		1	(42)
European Union		1	(33)
Finland		3	(22, 40, 43)
Greece		1	(42)
Italy		1	(44)
Netherlands		3	(18, 20, 45)
Poland		1	(20)
Portugal		2	(40, 46)
Spain		2	(20, 42)
United Kingdom		4	(20, 33, 40, 47)
Canada	North America	2	(48, 49)
United States		5	(7, 33, 50, 51, 52)
Israel	Asia	1	(53)
Japan		1	(54)
Singapore		2	(21, 55)
Turkey		1	(19)
Australia	Australia	1	(56)
New Zealand		1	(57)
**Participants selection method**			
Random sampling		15	(18, 20, 33, 40, 44, 46, 47, 49, 50, 51, 52, 53, 54, 56, 57)
Convenience sampling		8	(7, 19, 22, 41, 43, 45, 48, 55)
Snowball sampling		1	(42)
Stratified sampling		1	(21)
N/A^b^		1	(58)
**Survey method**			
Online questionnaire		21	(18–21, 33, 40–51, 52, 54, 55, 56)
Paper questionnaire/ handouts		2	(53, 57)
Interview/ group discussion		3	(40, 56, 57)
Meat product evaluation		5	(7, 18, 53, 55, 58)
**Cultured meat acceptance measures**			
Willingness to buy		11	(18, 21, 41, 42, 44, 47, 48, 49, 51, 52, 57)
Willingness to replace conventional meat		4	(21, 48, 51, 53)
Willingness to try/ eat		17	(18, 19, 21, 20, 33, 40, 41, 42, 43, 44, 45, 48, 50, 51, 52, 56, 57)
Willingness to reduce meat consumption		3	(20, 21, 42)
Willingness to replace other meat alternatives		2	(21, 48, 51)
Willingness to recommend to others		2	(19, 56)
**Sensory attributes as a primary outcome**			
Primary		6	(18, 43, 47, 53, 55, 58)
Secondary		18	(7, 19, 21, 22, 33, 40, 41, 42, 45, 44, 46, 48, 49, 50, 51, 52, 54, 57)
Inference		2	(20, 56)

a More than one response could be selected for a study. Thus, the total number of studies in each category may exceed the total number of included studies (*N* = 26).

b The corresponding study evaluated their samples instrumentally.

Although by definition of the search strategy all papers were related to the sensory evaluation of CM, the most common research question was only tangentially related to sensory evaluation: consumers’ “willingness to try/eat” (*N* = 17 studies) and “willingness to buy” (*N* = 11 studies) CM were the main acceptance measures used among the included studies. Some studies measured their participants’ willingness to replace conventional meat (*N* = 4 studies) or other meat alternatives (*N* = 2 studies) with CM and their willingness to recommend CM to other people (*N* = 2 studies) to support their studies. Through close reading of study objectives, we concluded that evaluating the sensory properties of CM was considered a primary outcome in 6 studies, while 2 studies inferred the importance of CM’s sensory properties. The majority (*N* = 18 studies) included sensory evaluation as a secondary outcome.

### 3.2 Hypothesized determinants for consumer acceptance of cell-cultured meat

The majority of the included studies examined consumers’ attitudes toward CM as a food product (i.e., instead of as a technology or a social innovation). Each paper opened by reporting positive actual or (frequently) potential impacts of CM (Table 3). Most proposed CM as part of a solution to reducing environmental pollution (*N* = 22 studies), minimizing the use of natural resources (*N* = 18 studies), promoting animal welfare (*N* = 19 studies), or promoting food security (*N* = 7 studies) along with fulfilling the increasing demand for animal proteins (*N* = 6 studies). CM was also said to have the same or possibly better nutritional value (*N* = 8 studies), to be safer to consume than (*N* = 11 studies), and to have the same sensory characteristics as conventional meat (*N* = 6 studies).

**Table 3:** Author signified values of cell-cultured meat that are emphasized in the introduction or hinted throughout the study.

Value framing	Number of studies^a^	References
Reduce environmental pollution	22	(18–22, 33, 40–44, 46–58)
Promote animal welfares	19	(18, 19, 22, 33, 40, 41, 42, 44, 45, 47, 48, 50, 51, 52, 54, 56–58)
Minimize the use of natural resources	18	(18, 20–22, 41, 44–54, 56–58)
Safer to consume than conventional meat	11	(18, 21, 22, 33, 40, 41, 42, 46, 47, 48, 53, 59)
As/ more nutritious than conventional meat	8	(18, 33, 41, 46, 47, 48, 50, 55)
Food security	7	(19, 21, 40, 41, 54, 56, 58)
Fulfill increasing demand of animal proteins	6	(18, 33, 41, 44, 56, 58)
Have the same sensory attributes/ experience as conventional meat	6	(18, 19, 49, 50, 52, 55)
Replace conventional meat	6	(33, 40, 42, 48, 51, 52)
Potential biodiversity loss	2	(20, 21)
Reduce meat consumption	1	(57)
Food source for outer space expeditions	1	(7)
Less familiar to the public	1	(43)

a More than one response could be selected for a study. Thus, the total number of studies in each category may exceed the total number of included studies (*N* = 26).

Despite these proposed possible benefits of CM from the studies’ authors, the factors measured in these studies and consumer responses often indicate alternative and more negative perspectives. Table 4 presents various factors that authors explicitly or implicitly hypothesized as impacts on consumers’ acceptance of CM. Common author-hypothesized determinants include views of animal welfare (*N* = 11 studies), concern over environmental impact of CM and the current systems of meat production method (*N* = 13 studies), familiarity and curiosity toward CM (*N* = 11 studies), perception of naturalness of CM (*N* = 6 studies), and psychological factors related to the individual such as food neophobia and disgust (*N* = 10 studies). The need to evaluate these determinants indicates that the authors of these studies are concerned with and well aware of alternatives to the positive narratives and impacts proposed in the introductions to these studies. Moreover, participants frequently mentioned concerns about CM’s health impacts, nutrition quality, or food safety (*N* = 18 studies) and, often, the sensory properties of CM (e.g., how it would taste, *N* = 18 studies), indicating that consumers echoed some of the concerns about whether CM will be a universally positive replacement for conventional meat products.

**Table 4:** Factors hypothesized (by authors) as determinants of cell-cultured meat acceptance among potential consumers.

Factors	Number of studies^a^	References
Health/ nutrition/ safety	18	(18, 19, 21, 22, 33, 40, 42–44, 46–54, 56)
Taste (tasty, have the same sensory experience as conventional meat)	18	(19, 20, 21, 22, 40, 41, 42, 44, 45, 46, 47, 48, 50, 53, 54, 56, 57, 58)
Environmental impact	13	(19–22, 40–46, 49, 51)
Animal welfare	11	(19, 21, 22, 41, 42, 44, 45, 48, 49, 51, 57)
Consumer knowledge about cultured meat	11	(18, 20, 42, 43, 45, 46, 48, 50, 51, 56, 57)
Psychology (food neophobia, disgust, belief in technology/ attitude)	10	(18–21, 40, 43, 45, 47, 48, 54)
Economic (price, income)	10	(19, 20, 40, 41, 42, 48, 49, 51, 56, 57)
Framing/ labeling	8	(18, 33, 44, 45, 46, 48, 50, 52)
Naturalness	6	(19, 40, 41, 45, 54, 56)
Demographic background	5	(22, 33, 49, 51, 57)
Convenience	3	(20, 40, 56)
Media coverage (including social media)	1	(22)

a More than one response could be selected for a study. Thus, the total number of studies in each category may exceed the total number of included studies (*N* = 26).

### 3.3 Perceived sensory experience of CM among tested consumers

Because most of these studies examined consumers’ holistic attitudes toward CM, it could be argued that understanding the sensory characteristics of CM was not the primary focus of the studies’ research designs. However, 18 out of the 26 included studies stated explicitly that sensory experience is a key determining factor for acceptance of this (or any) novel food product. When measuring participants’ willingness to accept CM, the questions were always framed to compare CM to conventional meat or meat products. This is implicitly a sensory comparison in the context of a consumer choice study. Unless the type of CM was specified (e.g., cultured seafood), CM was generally compared to a red meat or red meat product. In the reviewed articles, sensory experiences of CM among consumers were evaluated under two conditions: in the absence or the presence of actual products (Table 5).

**Table 5:** Methods used to obtain consumers’ perception of CM’s sensory experience with and without a physical sample.

How is opinion generated?	Framing theme	Number of studies^a^	References
Direct question of how they think CM would taste (no framing)		2	(21, 43)
Meat sample evaluation		5	(7, 18, 53, 55, 58)
	Others (e.g., economic, technology, sensory characteristics, societal, ethics)	19	(18, 19, 33, 40–42, 44–51, 52, 54, 56, 57)
Positive framing	Health/ safety	13	(18, 19, 33, 40, 42, 44, 46–51, 56)
	Animal welfare	9	(19, 41, 42, 44, 45, 48, 49, 51, 57)
	Labeling	8	(18, 33, 44–46, 48, 50)
	Environmental benefits	6	(19, 41, 42, 44, 49, 51)
	Naturalness	5	(40, 41, 45, 54, 56)
	Meal setting & placebo panel	2	(18, 46)

a More than one response could be selected for a study. Thus, the total number of studies in each category may exceed the total number of included studies (*N* = 26).

#### 3.3.1 Sensory characteristics in the absence of product evaluation

The majority of the included studies (*N* = 21 studies) did not involve subjects evaluating any actual meat samples, conventional or CM. The studies instead examined consumers’ *expectations* of CM sensory experiences by provoking participants’ imaginations: 2 studies asked consumers directly about their expectations for CM taste, and another 19 asked this question after providing an information frame (by words and/ or images) that was meant to affect consumers’ expectations. A number of studies reported that participants did not believe that CM would be as tasty as conventional meat and CM products were described as unappealing, disgusting, and were not considered as meat (*N* = 9 studies). Moreover, when compared in a variety of preference-ranking methods to other meat alternatives (e.g., soy- and other vegetarian products, insect-based proteins), CM was often placed at the bottom of the preference rankings along with insect-based alternatives, and consumers seemed to prefer the concept of plant-based alternatives (*N* = 7 studies).

In a large minority of studies (*N* = 8), consumers responded positively toward CM as a meat product. Typically, the positive attitude was in response to positive information framing pertaining to product labeling (50), safety and health benefits (44), impact on the environment and animal welfare (45), or perceived naturalness (40) - especially in regards to the production process. The overall measure of acceptance was mainly based on increases in participants’ willingness to try and willingness to buy or pay for CM along with individual perception ratings of tested factors listed in section 3.2 (*N* = 6). Decrease in disgust perception of CM and increase in perceived similarity of CM with conventional meat products were also considered positive responses (*N* = 4). Introducing CM in familiar meals and providing placebo samples to provoke the idea of sensorial similarity of cultured to conventional meat was reported as effective in increasing tested participants’ acceptance of CM (*N* = 2 studies).

#### 3.3.2 Sensory characteristics based on product evaluation

Actual evaluations of some sort of CM-relevant product were conducted in only 5 out of the 26 studies, with 1 study using a placebo sample and 4 studies evaluating an actual CM sample. Commercially produced beef was used in the placebo sensory testing while the CM samples were made in-house by the researchers. A detailed summary of each study’s method and key findings is presented in Table 6.

**Table 6:** Summary of product evaluation of meat products in the included studies (*N* = 5 studies).

Papers Reference	Product	Sensory evaluation method	Key findings
Rolland et al. (18)	Commercial beef burgers	A wanting/ liking test was conducted on identical commercial beef burgers. Samples were presented to panelists as ‘conventional’ and ‘cultured’. ‘Cultured’ hamburgers were served in a smaller portion than ‘conventional’. Participants (N = 193) rated the hamburgers’ appearance, color, smell, tenderness, and juiciness.	High acceptance of cultured meat among panelists when the known information about cultured meat is positive and supported by a favorable tasting experience. Overall, participants perceived cultured meat as safe and appropriate food.
Benjamison et al. (7)	Cultured goldfish	Fried fish filets were evaluated for aroma and appearance by 4 panelists.	Cultured fish reacted to the cooking process as would fresh fish. Panelists perceived the product acceptable as food despite the absence of tasting.
Ong et al. (55)	Cultured pork [porcine myoblast on texturized soy protein (TSP) and jack fruit-containing scaffold (JFS)]	A single-blind test was conducted on a photo of (created) cultured pork grown on JFS or TSP scaffold Participants (N = 78 university students) rated their attitudes toward the product.	Pan fried JFS-cultured pork showed meat-like browning behavior and potentially shelf-stable meat-like color. The use of JFS improved participants’ perception of the product by more than 8%.
Lee et al. (58)	Fish gelatin/ agar (GA)-coated textured vegetable protein (TVP) scaffold with mouse blast as model meat cells	Scaffolds made of TVP, GA-coated TVP, and a GA-coated TVP with mouse myoblast were compared to commercial beef cuts (chuck, tenderloin, and brisket) for texture, flavor, and taste. Evaluations were done using analytical instruments.	Cultured meat’s texture, flavor, and taste implied as comparable to that of slaughtered meat due to the synergistic effect between the myoblast and scaffold.
Pasitka et al. (53)	Cultured chicken (Mixed-breed chicken cultured adipocyte-like cells combined with extruded soy protein)	Panel 1: cultured chicken was served as a meal and rated for overall impression, flavor, texture, aroma, and overall experience of the product (N = 13 participants).	Overall, participants found the cultured chicken dish acceptable (average likelihood of 8/10 to replace farm-raised chicken with cultured chicken). Sixty seven percent of the blind-tasting participants preferred cultured chicken over the soy-based alternative.
		Panel 2: cultured chicken tasted alongside soy-based chicken and rated for their texture and flavor in a blinded-test (N = 30 participants).	
		Panel 3: cultured and soy-based chicken in comparison to farm-raised chicken breast. Participants (N = 13) were asked to rate the general flavor, texture, aroma, and overall experience.	

Rolland et al. (18) conducted a sensory evaluation using placebo samples to gage consumers’ likely response to CM with sensory qualities identical to conventional meat. In this experiment, Rolland et al. (18) evaluated participants’ initial responses to the concept of CM and then measured changes to those responses after presentation of one of three positive information framings on the *societal, personal,* or *sensory/quality benefits* of CM, and finally, measured actual liking and some basic sensory-quality measurements in response to evaluation of a placebo CM sample. In this case “placebo” means participants did not evaluate actual CM at any point in the study. Instead, cooked commercial beef burgers (patties) were served labeled as ‘cultured’ and ‘conventional’ in different sizes - ‘cultured’ burger was served as a smaller piece to indicate limited availability. Rolland et al. (18) found an initial positive perception of CM among participants who claimed to “know exactly what CM is” (p. 8). Participants who had never heard of CM or had heard of it but were unfamiliar with it experienced a greater increase in acceptance toward CM. The placebo sensory evaluation also increased consumer acceptance: after tasting the purported CM, a higher score in willingness to taste CM was observed, participants reported willingness to pay a premium price for CM (on average 37% higher price than conventional meat), and positive judgment on the taste of CM was observed.

Benjaminson et al. (7) reported the first successful attempt to grow CM in the form of goldfish tissue-explant “fish fillets.” The resulting product was then cooked and evaluated for its aroma and appearance (but not taste) by 4 employees of the lab without sensory-evaluation training. Cultured fish was reported to be as easy to harvest and to react the same way when cooked as conventional fish filets would. From a sensory point of view, cultured-fish filets were reported to be glistening, firm, and odorless (7).

In the last several decades, direct tissue-explant methods for growing CM have been largely superseded by scaffold-based methods, which is reflected in the more recent sensory-evaluation studies [*N* = 3; (53, 55, 58)]. Ong et al. (55) used jackfruit/textured soy-protein (TSP)-based scaffolds to grow pork cells. This method was shown able to mimic seared beef’s shrinking and color-changing behavior (although, not seared pork’s) which the authors implied to as an “indicat[ion of] its utility to mimic cooked meat” (p. 5). Ong et al. (55) conducted a between-subject, visual sensory evaluation, asking each of the 2 groups of participants to evaluate a picture of either TSP-scaffolded (*N* = 38 untrained participants) or jackfruit/TSP-scaffolded (*N* = 40 untrained participants) cultured pork. Based on consumer evaluation of pictures of the grown cultured pork, the meat-like mimicry of the jackfruit/TSP significantly scaffold improved participants’ perception of CM products by more than 8% compared to the TSP-based scaffold (55).

Lee et al. (58) also investigated the effects of novel scaffold on sensory-relevant characteristics of CM products. The authors created a TVP/fish gelatin-based scaffold and grew mouse cells as their CM model. Lee et al. (58) then instrumentally analyzed for color (colorimeter), texture (texture profile analysis in comparison to multiple beef brisket, chuck, and tenderloin), flavor (GC–MS), and taste (electronic tongue). The authors described their product as having a similar texture to a beef tenderloin, although the product was based on mouse cells. Flavor analysis of the cooked product confirmed the presence of common Maillard-browning produced aroma compounds, namely acetophenone, 2-ethyl-1-hexanol, nonanal, octanal, and nonanol (58), although it is important to note that these compounds are not the only or even the most characteristic products of Maillard browning, and that Maillard browning can occur whenever proteins and sugars are heated together, not only in meat products (60). Moreover, the listed compounds were not determinants of meat sensory characteristics as most key meaty aroma compounds from Maillard reaction are heterocyclic (61, 62). Taste analysis showed that compared to a beef-brisket sample, the cultured mouse sample had similar (predicted) bitterness with lower sourness and higher umami values. The perceived aftertastes [“the taste that remains on the tongue after completely swallowing the food”; (58), p. 38242], namely astringency, bitterness, and umami were similar to the tested beef-brisket.

The only study that conducted sensory evaluation with human subjects that involved actual tasting of a real CM product was published very recently by Pasitka et al. (53). Sensory evaluation by untrained panelists was conducted in 3 different studies which evaluated overall sensory characteristics of a hybrid plant-protein/cell-cultivated chicken product (*N* = 13 participants), a preference test comparing this hybrid-CM product against a soy-based chicken (*N* = 30 participants), and a preference test comparing the hybrid-CM, the soy-based chicken, and conventional chicken (*N* = 13 participants). Based on the (forced-choice) preference test, most participants (67% of 30 participants) favored cultured chicken over soy-based chicken in terms of sensory attributes. In the first sensory evaluation, when participants (*N* = 13) were asked “how likely are you to replace your meat choice with this (cultured chicken) product?” [(53), p. 41], “the average likelihood stated by participants was 8/10” [(53), p. 41]. The cultured chicken also showed remarkable similarities to conventional chicken in sensory attributes, including taste.

## 4 Discussion

The studies identified for this review were almost entirely affective consumer studies, with no analytical sensory evaluations (63). Although the eligibility criteria were not designed to identify solely affective studies (see section 2.2), the prevalence of affective studies speaks to the relation between products’ sensory characteristics and consumers’ decision-making when it comes to any food. This can be further explained by considering the complimentary relationship between sensory science and consumer science in the analysis of “product micro lifecycle” from product purchase to consumption (64). Consumer science focuses on explaining consumers’ choice based on psychological stimuli such as product information and past experiences while sensory science explains consumption based on psychophysics—a combination of physical stimuli and human perception (64). Therefore, understanding the concept and actual identity of CM is necessary.

### 4.1 Sensory perception based on imagined or expected products

The main discussion in this section revolves around the CM sensory characteristics claim and consumer acceptance based on evaluation of CM as an idea (positive information framing), not an actual product evaluation. As shown in the results (section 3.3.1), a majority of studies’ respondents had negative expectations of CM as an imagined product, and particularly of its sensory qualities. Novel-product unfamiliarity was hypothesized as the main reason for consumer skepticism toward CM. For example, according to Weinrich et al. (65), pre-knowledge of a product was a mediator between one’s demographic background and attitudes toward the product. On a similar note, Lin-Hi et al. (66) considered CM as a “radical innovation in food sector” - an innovation that radically breaks with familiar logic and habits - which they hypothesized explained the tendencies for consumers’ skepticism toward this product. The implication is that this skepticism manifested in multiple ways including perceptions of disgust (67), and perceptions that CM is more unsafe and unnatural, leading to further rejection of new food products (33, 67–70).

In order to determine whether unfamiliarity, skepticism, or neophobia might drive negative expectations around CM, information framing was used in many of these studies to determine if different frames increased acceptance. Various positive framings from labeling to the use of ‘placebo’ products were conducted and, overall, these all tended to improve expected consumer acceptance toward the product when properly directed (section 3.3.1). Positive framing focused on aspects such as product safety, health benefits, and environmental sustainability was found to increase consumer acceptance. This aligned with suggestions from many studies to highlight the key drivers of consumer acceptance namely safety and health benefits as well as showing that CM is a natural product that resembles conventional meat (18, 71–73). These framing approaches to increasing acceptance may be necessary as some studies reported that while some consumers (initially) were willing to support CM because of the benefits to animal welfare and the environment (65, 70), many consumers are not actually aware of these adverse environmental and animal-welfare impacts of the conventional system of producing meat. Therefore, in many studies these frames may be simultaneously informing consumers of a problem and providing CM as a solution (13, 74).

It is important to note that all 26 studies reviewed framed CM positively, regardless of the specific frame. The selection criteria for the scoping review were not designed to select papers with a particular position on this sometimes-controversial biotechnology (75), so this result is itself noteworthy. Since many of the study authors are apparently invested in the potential of CM as a meat-production method, this preponderance of positive framing may not have been the *explicit* intention of authors of the reviewed studies, either. However, since there have apparently been no sensory- or consumer-evaluation papers that investigated the effect of a *negative* framing on CM, this may be a source of bias in the literature, particularly confirmation bias.

As reported by Ryynänen and Toivanen [(22); see also (76)], based on their exploration of the role of written and online media in framing and presenting CM, most articles highlighted only benefits of CM, and presented the only challenges for CM as the current high cost of production and the possible imperfect reproduction of the sensory characteristics of conventional meat. This absence of real critical framing may create a critical knowledge gap, since CM cannot truly be said to be risk-free. For example, based on their intensive review, Bhat et al. (4) predict CM to be more likely to have a substrate contamination risk from the growth media, in contrast to the bacterial contamination from processing that is a problem for conventional meat. An overview by Broucke et al. (77) claims that the “addition of compounds and solutions like sera, growth-hormone factors (GHF), (bovine serum) albumin (BSA), and transferrin, withhold an additional risk due to possible introduction of harmful or pathogenic agents, especially in the case of *in vivo* gained animal sera (mostly fetal, but also new-born or adult source). Examples of possible contamination are prions, bacteria (including mycoplasma), and viruses (e.g., hepatitis virus)” (p. 7). Risks may also arise within the cell-handling and -cultivation process; not only additional contamination, but also potential genetic drift that can cause unintentional genome alteration of the cultured cells (78). Furthermore, it is possible for adult stem cells to become malignant in long-term culture (79). The effect of these more negative counter-narratives on consumer acceptance have yet to be explored, thus it may be premature to conclude that only positive framing has an effect on consumer willingness to buy/eat CM and their perceptions of its quality.

### 4.2 Sensory characteristics based on actual product evaluation

Despite the many promises and the insistence in the literature that CM will have sensory characteristics equivalent to conventional meat (18, 80, 81), there was very limited evidence of the actual sensory characteristics of CM. In this review, (meat) product evaluation was only found in 5 studies, with 1 conducted on a “placebo” consisting solely of conventional meat, and 4 on CM (see section 3.3.2). Overall, these studies did conclude that the evaluated CM closely resembled its conventional counterpart in both sensory characteristics and reaction to cooking. These studies speak to the potential for CM products to ultimately resemble their conventional equivalents.

However, the claim that CM products will always have, or even currently have, the same sensory characteristics as conventional meat products is not well-established in this current literature. Three main issues are as follows: (1) only one study reports an actual sensory evaluation with tasting of CM products; (2) in all studies excepting Rolland et al. (18) (which did not actually involve CM products), evaluations were conducted with unacceptably low numbers of human panelists (this standard is described with more detail in section 4.2.2); and (3) the CM products evaluated were not comparable to their target, conventional equivalents.

#### 4.2.1 Lack of product tasting

Almost all studies did not involve actual tasting of a product, CM or otherwise. Lee et al. (58), Pasitka et al. (53), and Rolland et al. (18) stood out among the actual sensory evaluations for reporting details about taste and flavor in CM products (see section 3.3.2). Among these three studies, only the placebo panel by Rolland et al. (18) and the CM sensory evaluation by Pasitka et al. (53) included actual human-subject evaluation by taste of any product.

The result of the Rolland et al. (18) placebo panel (section 3.3.2) was considered a notable success in the acceptance of CM products, not only by the authors, but also by many others who have cited these results as proof that consumers will accept CM (59, 68, 82, 83). Compared to a past consumer liking test investigating novel food technologies by Tan et al. (84)—participants were served novel foods such as lamb brain, frog meat, or meal-worms burger and rated the products as inappropriate for food—Rolland et al. (18) results with a sensory panel evaluating placebo products found that ‘cultured’ hamburger was considered to taste slightly better than ‘conventional’, and to therefore be acceptable as a substitute. The authors also noted that among the 4 acceptance questions they used, “…the willingness to taste cultured meat had a much higher score than the responses to the other questions” [(18), p. 13]. Interpreting this statement, the authors hypothesize “[a]s perceived danger is a major determinant for willingness to taste novel foods [26], this suggests that participants did not consider cultured meat dangerous” [(18), p. 13]. Thus, “…a cultured meat hamburger is considered an appropriate food [by participants] when its sensory features are equivalent to conventional meat” [(18), p. 13].

Looking closely at the context of the Rolland et al. (18) study, however, these results are scarcely indicative of consumers’ acceptance of CM. Rather than perceiving CM as safe to eat, the high willingness to taste CM score could as plausibly be interpreted to reflect participants’ curiosity about the product, considering it was not yet at all available in the market, and the notoriety of some of the study’s authors, who were responsible for the first televised tasting of CM hamburger (85, 86). Since the study was *in vitro* it also does not establish whether consumers would be willing to continue consuming the product (87, 88). Furthermore, it should be emphasized, as conventional hamburger was the only sample presented in this study to taste, this study did not prove anything about the sensory characteristics or acceptability of CM; instead, it showed how much participants like the taste of conventional meat. The results are only generalizable to CM if it does indeed have exactly the same sensory characteristics of conventional, beef hamburger.

The study by Lee et al. (58), although included in our review because of the detailed attention to sensory characteristics of its CM sample, did not use human senses to evaluate the product. Instead, they use instrumental analyses to *predict* flavor, taste, and texture characteristics. Although created to model a human tongue, the sensor performance of an electric tongue differs with respect to sensitivity, selectivity, and detection limit for the compounds of interest (89). Regarding flavor as measured by volatile aroma compounds, the absence of furans, pyrazines, oxazoles, and other essential sulfur-containing flavor compounds in the final product are concerning, and may indicate that the CM will not have a flavor equivalent to conventional meat, since these are key aroma compounds for red meats (58, 90, 91). In the absence of any human sensory evaluation, these results should not be treated as indicative of the “true” sensory experience of CM.

The hybrid (plant and cell based) chicken product evaluated in the study by Pasitka et al. (53) is currently the only CM product currently reported in the literature to have undergone sensory evaluation for flavor by human subjects.

#### 4.2.2 Low power sensory studies

Sensory evaluation focuses on person-product interaction; it requires an interaction between a person and a stimulus (63). Colloquially, sensory evaluation uses human subjects as “instruments” to determine the analytical or affective characteristics of a (food) product. Typically, sensory evaluation methods are broken down into three broad categories: discrimination tests (“are their perceptible differences among samples?”; require trained or untrained panelists), descriptive tests (“what are the perceived sensory differences among the samples?”; require trained panelists), and affective/hedonic tests (“how are these samples liked by different subjects?”; require untrained panelists) testing.

Among the included studies, the studies by Benjaminson et al. (7) and Pasitka et al. (53) were the only ones to involve direct person-to-CM evaluation. It is important to recall that Benjaminson et al. (7) did not allow subjects to taste the samples. Both studies based their results on a number of subjects that would be universally considered too low for statistical power by the standards of sensory science (see section 3.3.2). Both studies did not specify the specific objective(s) or research questions that were addressed by conducting sensory evaluation, but based on their results, the authors seemed interested in identifying similarities between their CM and its conventional counterpart as well as proving its acceptability among consumers. The recommended number of panelist in an affective study depends on several factors, including the expected quantitative differences among products, the specific research question, the method of collecting data, the desired population to which the results should generalize, and the complexity of the products themselves (92). To achieve a proper predictive validity, it is typically recommended to have 24–40 panelists for a simple difference test and 50–100 consumers for a hedonic test without post-hoc segmentation (63, 92, 93). Unfortunately, Benjaminson (*N* = 4 panelists) and Pasitka (N ≤ 30 panelists, depending on sub-study) simply did not include sufficient panelists in their studies.

Furthermore, within an affective test, Pasitka et al. (53) asked their untrained panelists to measure specific sensory attributes such as sweetness, savoriness, saltiness, aftertastes, chicken flavor, and other attributes (Table 6). This type of sensory-evaluation test should be done through descriptive sensory methods, which require 8–12 well-trained panelists: individuals that have been trained to evaluate reference standards and reach an objective, within-group consensus about the meaning of terms like “chicken flavor” (63). The statistical power of descriptive methods even with such a small number of panelists is typically justified by the reduction of variance through this calibration (training). Furthermore, recent research has shown that even “simple” terms like sweetness are not suitable for evaluation by untrained panelists (94). Thus, the conclusions about specific sensory attributes based on very small, untrained panels as in the study by Pasitka et al. (53) are unlikely to be reliable.

#### 4.2.3 Non-equivalent sample evaluation

Of the few studies that actually produced CM samples for evaluation, two studies evaluated samples that do not correspond to meat that people usually consume, namely cultured goldfish and cultured mouse. The creation of a goldfish filet by Benjaminson et al. (7) was the first success story for the creation of cultured fish. Although the process was not yet practical from a yield standpoint at that time, the authors stated that they had successfully “... addressed fundamental *in vitro* skeletal muscle growth parameters” and that “…the yield versus cost calculation projects a favorable outcome provided sufficient research effort and resources become available” [(7), p. 887]. Of course, humans do not typically consume goldfish, so this work on a model species was meant to be a stepping-stone toward the production of CM fish from actual food species. It is not unreasonable to observe that this study was published more than two decades ago; by now other labs working on cultured fish should surely have been able to improve on or at least replicate results of Benjaminson et al. (7). Unfortunately, this optimism does not seem to have been realized; not only is cultured fish not in the market as of this time of writing, but no further record of evaluation on cultured fish product(s) has been published for inclusion in this review.

The creation of CM that mimics the texture of beef tenderloins is certainly worthy of note (58). However, there is not yet evidence that this approach can succeed using cells from animal species that are more commonly consumed than mice. This consideration is not merely a quibble, considering the different genomic resources between species which confine cell differentiation to result in tissues that are species-specific (95, 96). Thus, being able to produce ‘cultured beef tenderloin’ from mouse cells does not guarantee that this approach will be successful for the creation of products with, for example, cow cells.

### 4.3 Realities of CM production

The majority of papers included in this review (*N* = 24 studies) present CM as an entirely positive, transformational biotechnology. This is emphasized especially in studies that communicated these concepts to participants as framing (*N* = 18 studies). In framing studies, participants are asked to believe that the given, invariably positive frame about CM is an unproblematic truth. These frames are typically consumer-appropriate versions of the arguments given in support of CM as a technology in the literature (*N* = 18 studies; examples in Table 5 in “positive framing”). However, as reported in an increasing number of studies, these arguments in favor of CM are exactly that—arguments, not inevitable truths (77, 88). Predicting the future development of CM as both a process for producing a food and as a consumer product has turned out to be considerably more complicated.

Typically, arguments in the papers reviewed here imply that CM is isomorphic with conventional meat but produced in a more sustainable way. However, as long as CM has continued to be a hypothetical product, this claim has not been supported by enough evidence. CM typically is argued to be more sustainable because it would cause less environmental damage—requiring fewer natural resources and less land use, and causing lower greenhouse-effect gas-emission—than conventional forms of livestock production for meat (2, 4, 6). However, those statements are typically based on theoretical projections with large uncertainties (97, 98). In addition, although *in vitro* CM production may require lower agricultural inputs, including land use, it would require more intensive energy use in return. Lynch and Pierrehumbert (99) also reported that, although cattle farming has grater peak (global) warming effect, the warming effect would not persist nor accumulate under reduced ‘farming’ system as would *in vitro* meat production. While CM production’s benefits in terms of sustainability are likely to be much more complex and contingent, the papers reviewed here presented CM production only in terms of maximal, unalloyed benefits, both in argumentation (*N* = 24 studies) and in experimental design (*N* = 18 studies).

As for CM being exactly the same as ‘real meat’, the burger grown in Professor Mark Post’s laboratory and presented in 2013 has been, to this date, the only consumed ‘pure (cultured) meat’ in real life. According to the three tasters, “…the burger was dry and a bit lacking in flavor” (85) in which one of them described “the bite [texture] feels like a “conventional hamburger” but that the meat tasted “like an animal-protein cake.”” (85). The recently sold cultured chicken and fish products are a hybrid of animal cells and plant-based materials (100). This reality is reflected in this review; the only CM product that was evaluated by taste was a hybrid product of soy protein and chicken cells and not *just* ‘animal meat’ (53) as is typically presented in the framing and narratives documented in these studies.

Beyond the challenge of developing a CM product that can be an acceptable sensory substitute for conventional meat, high production costs have remained a hurdle. Proponents of CM have claimed that Moore’s law - formulated for microprocessors, and arguing that the cost of production of novel products will always reduce exponentially over time - would apply to CM (88). Moore’s law has, however, not typically been applied to biological systems, which are the basis of CM production, due to the complexity and unpredictability of biological events and the mechanisms behind it (88). Ten years ago, a five ounce burger costed over $300,000 to make (85, 101) and so far, CM products have been sold for about $18 per meal—a loss regarding which producers were not willing to share further details on (102). Thus far, reframing CM as a plant/animal-cell hybrid product seems to be more realistic both from a product-formulation and a production-cost point of view. Using cell cultures as flavorings of a plant-based meat is what most CM producers are moving toward (102). The future of CM has once again been proven unpredictable, suggesting the importance of sober and realistic interpretation of the literature in order to avoid bias and overexcitement.

Other than to further understand potential consumer attitudes toward CM, many consumer acceptance studies were directed to ensure the *increase* in consumer acceptance of CM (34–36). For example, after determining drivers for consumer acceptance and rejection of CM, the authors suggested ways to market CM such as framing CM as a solution to the existing food safety problems (68) and portraying CM as more natural, favorable, and addressed consumer concerns about the technology could improve consumer perceptions of the product (70). A similar pattern was also found in more recent publications (66, 72). This indicated that increasing consumer acceptance is the main interest of CM proponents which could be a critical source of bias throughout literature in this area.

### 4.4 Researcher investment in CM as an idea

The intense promotion of CM as a world-changing biotechnology makes research around this topic prone to bias. To investigate this, conflict of interest statements, study supports (source of funding, panelist, and samples), and authors affiliations were examined (Appendix 5). Based on the results, the panelists and sample sources were unlikely to be a source for bias/conflict of interest. This was because the studies used random participants from a crowd sourcing website, conducted national/cross-country survey, or obtained random internal participants such as college students. Only the study by Benjaminson et al. (7) acknowledged using panelists who were their own lab members. As for sample sources, almost all studies that conducted a sensory panel made their own sample. Only one study bought commercial samples [hamburger; (18)]. Further details can be seen in the Supplementary material.

Possible sources of bias were sought in the conflict of interest statement, author affiliations, and funding sources (Appendix 5). Eight of the included studies did not include a conflict of interest statement while most studies (*N* = 17) declared no conflict of interest. Ong et al. (55) was the only study that declared potential conflict of interest. Ong et al. (55) declared that portions of the reported research has been submitted for a patent (patent application no. PCT/SG2020/050432) to two of their authors, Shujian Ong and Hanry Yu. Furthermore, both Shujian Ong and Hanry Yu were affiliated with Ants Innovate Pte Ltd., a Singapore deep tech start-up that focuses on developing cultivated whole meat cuts. Studies’ funding sources showed that many included studies were funded through a government grant and/or internal funding (*N* = 12 studies), from independent-nonprofit organizations (*N* = 12), and from the CM industry (*N* = 2 studies). Upon examining the authors’ affiliations, almost all authors were university affiliates, with only a few independent or industry researchers (*N* = 3 studies). These 3 studies included at least an author that is affiliated with a CM industry: Ants Innovate Pte Ltd., Believer Meats, and Mosa Meat (Appendix 5).

While the development of cultured meat is happening across public research labs and private industry, most of the reported success in producing CM products is in private industries such as Upside foods, Good Meat Inc., Scifi foods, and Blue Nalu (100, 103). With CM formulation becoming companies’ best-kept secret, perhaps for this reason the real sensory characteristics of CM are still unreported in the scientific literature. In this review, CM products that were evaluated were all made in-house by the research teams. This implies that CM products produced by industry were not made available for independent assessments. If CM is only developed by a handful of individuals that are likely proponents of this product, implicit bias of reporting positive results is very likely to take place; it is very unlikely that negative results will be published at all. In fact, the known consumer related studies and information given to the media are mostly the success stories of CM development (22, 76).

## 5 Limitations

This scoping review focused on identifying proof of the sensory characteristics of cell-cultured meat (CM), not to identify the consumers’ responses toward CM or judging the product of CM. Thus, the results should be interpreted within this context, not necessarily in terms of the larger feasibility of making high-quality CM acceptable to consumers or even the possible *future* sensory characteristics of CM products.

In terms of data collection, this scoping review was restricted to publications in three agriculture-based databases. Considering the extremely active nature of publications around the topic of CM, it is likely that some manuscripts are not retrieved from our initial search. For example, while studies by Chriki et al. (104) and Liu et al. (105) were not captured by our search strategy, they were brought to our attention after article submission. While these articles would not significantly change our conclusions about the state of the field—both asked consumers to hypothesize about how “tasty” cultured meat would be—they are an example of the active and rapid state of the discourse. A different result might be obtained if more than three databases were included or done in multidisciplinary databases (e.g., sociology, psychology, and communications) or related disciplines such as engineering. Furthermore, the review was restricted to publications written in English language. This means that the findings may not reflect the body of literature in other languages. The scoping review was designed to include the sensory characteristics of CM in general which was reflected in the absence of use of specific meat terms (e.g., “cultured seafood,” “cultured chicken”) in the search strategy. Thus, the results of this review should not be used to indicate a very specific type of CM.

As for the review method, the citation chasing approach could be improved. Instead of only backward citation chasing, a more thorough citation chasing could be done which includes checking all references in the included articles. Finally, data extraction was conducted by only one author with a second author as a reviewer (spot checking); although a valid approach, having more expert opinion in the process would further reduce the chances of misinterpretation.

## 6 Conclusion

Cell-cultured meat (CM) has been widely introduced as *meat produced in a more sustainable way*, implying it is better for the environment and will have the same sensory characteristics of conventional meat. Using structured, best-practice, scoping-review methods, the current state of knowledge regarding CM’s sensory characteristics was surveyed, with a focus on both the directly known attributes and the methods used to identify those attributes in the literature.

In the literature CM sensory-attribute characterization was performed regardless of product availability where a majority (*N* = 19 studies) of the included studies use positive framing to provoke stated preferences of CM among consumers and only a few (*N* = 5) studies conducted anything that could be characterized as an actual product evaluation. All reviewed studies demonstrated some possible weaknesses for drawing certain conclusions about the sensory characteristics of CM, namely: not actually tasting CM, low statistical power, or evaluation of unrealistic CM samples. Therefore, we must conclude that there simply is not currently evidence of the strong claim found throughout the larger literature that CM will have the same sensory characteristics as conventionally produced meat products. What the large minority of the included studies did show were possibilities to create cell-cultured products (CM) that mimic the color, texture, and response to cooking (color change and shrinking) of their conventional counterparts.

In conclusion, with the strong flux of advancements and reformulations currently ongoing around CM production, the sensory characteristics of cultured meat remain a mystery. Based on what is known, it is fair to state that CM still has a long way to go before achieving the exact sensory characteristics of the conventional meat that is in the market today. Based on the reviewed studies, it seems that the future of CM may be a hybrid product of cultured animal cells and plant proteins, which may achieve desirable sensory characteristics but will almost certainly not be exactly equivalent in sensory characteristics to conventional meat. Outside of the primary focus of this review it was observed that recent developments in CM are reported more in the mass media than in the peer-reviewed literature. Thus, media studies, communications studies, and sociological research in this area could contribute to clarifying what is expected and known about the actual sensory characteristics of CM. For the moment, from this review it is only possible to conclude that CM may someday succeed in producing a product with desirable and meat-like sensory characteristics; the guarantees and claims currently being made are not well-based in the peer-reviewed literature.

## Data availability statement

The original contributions presented in the study are included in the article/Supplementary material, further inquiries can be directed to the corresponding author.

## Author contributions

KT: Conceptualization, Data curation, Formal analysis, Methodology, Writing – original draft, Writing – review & editing, Investigation. CC: Conceptualization, Methodology, Writing – review & editing. SO’K: Writing – review & editing. JL: Conceptualization, Data curation, Methodology, Supervision, Writing – review & editing, Resources, Writing – original draft.
