# Supplementary material for: A taste of cell-cultured meat: a scoping review
Source: Front Nutr. 2024 Jan 23;11:1332765. doi: 10.3389/fnut.2024.1332765 (PMC10844533; doi:10.3389/fnut.2024.1332765)
Supplement: Supplementary file 1 [file Table_1.DOCX]

**Supplementary materials:**

# **Appendix 1: Search strings to identify literature related to cell-cultured meat sensory characteristics from 3 databases (Web of Science, Food Science and Technology Abstracts, and Center for Agriculture and Bioscience International Direct.**

| **Database** | **#** | **Search strings^a^** | **Limits**  **(including built-in filters)** | **Initial yield^b^** | **Final yield^c^** |
| --- | --- | --- | --- | --- | --- |
| Web of Science | 1 | TS=("culture? meat" OR "cultivate? meat" OR "lab-grown meat" OR "cell-based" OR "clean meat" OR "in-vitro meat" OR "artificial meat" OR "synthetic meat" OR "cell* meat") |  |  |  |
|  | 2 | TS=(accept* OR attitude OR preference OR perception OR fram* OR willingness OR awareness OR liking) |  |  |  |
|  | 3 | TS=(taste OR texture OR flavo?r OR appearance OR look OR sensory) |  |  |  |
|  | 4 | #1 AND #2 AND #3 | Publication year: 2000 – 2023; Document types: article | 262 | 386 |
|  |  |  |  |  |  |
| Food Science and Technology Abstracts | 1 | (TI ( "culture# meat" OR "cultivate# meat" OR "lab-grown meat" OR "in-vitro meat" OR "cell-based meat" OR "cell* meat" OR "artificial meat" OR "synthetic meat" OR "clean meat" ) OR AB ( "culture# meat" OR "cultivate# meat" OR "lab-grown meat" OR "in-vitro meat" OR "cell-based meat" OR "cell* meat" OR "artificial meat" OR "synthetic meat" OR "clean meat" ) OR KW ( "culture# meat" OR "cultivate# meat" OR "lab-grown meat" OR "in-vitro meat" OR "cell-based meat" OR "cell* meat" OR "artificial meat" OR "synthetic meat" OR "clean meat" )) |  |  |  |
|  | 2 | (TI ( accept* OR attitude OR preference OR perception OR fram* OR willingness OR awareness OR liking ) OR AB ( accept* OR attitude OR preference OR perception OR fram* OR willingness OR awareness OR liking ) OR KW ( accept* OR attitude OR preference OR perception OR fram* OR willingness OR awareness OR liking )) |  |  |  |
|  | 3 | (TI ( taste OR texture OR flavo#r OR appearance OR look OR sensory ) OR AB ( taste OR texture OR flavo#r OR appearance OR look OR sensory ) OR KW ( taste OR texture OR flavo#r OR appearance OR look OR sensory )) |  |  |  |
|  | 4 | #1 AND #2 AND #3 | Scholarly (peer-reviewed) journals; Publication date: start year 2000 – End year 2023 | 33 | 55 |
|  |  |  |  |  |  |
| CAB Direct | 1 | ("culture$ meat" OR "cultivate$ meat" OR "lab-grown meat" OR "in-vitro meat" OR "cell-based meat" OR "cell* meat" OR "artificial meat" OR "synthetic meat" OR "clean meat") |  |  |  |
|  | 2 | (accept* OR attitude OR preference OR perception OR fram* OR willingness OR awareness OR liking) |  |  |  |
|  | 3 | (taste OR texture OR flavo$r OR appearance OR look OR sensory) |  |  |  |
|  | 4 | #1 AND #2 AND #3 AND | Published between: 2000 and 2023; Document type: Journal article | 22 | 50 |

^a^TS: Topic search (Searches title, abstract, author keywords, and Keywords Plus; ?: To retrieve words with the replacement of 1 character [Example: wom?n includes women, woman] in Web of Science; #: Wildcard. adding the # in places where an alternate spelling may contain an extra character [Example: colo#r to find all citations containing color or colour] in Food Science and Technology Abstracts; $: To retrieve words with variant zero or 1 character [Example: colo$r to find color and colour] in CAB Direct; *: Truncation – to retrieve words with variant zero to many characters [Example: disease* will include diseases, diseased, diseasing, diseasedness etc.].

^b^Initial yield represents the number of articles retrieved from each database through the first search on January 12^th^, 2023.

^c^Final yield represents the number of articles gathered throughout the monthly search (January 12^th^, 2022 – June 11^th^, 2023).

# Appendix 2: Data extraction form (with some data extraction options truncated) for the scoping review of cell-cultured meat sensory analysis in current (mid 2022) literature.

GENERAL INFORMATION

1. What is the paper type (type of study)? (select one that apply)
   - Research
   - Review

If it is a review paper, stop extracting and continue with the next paper.

1. Insert the article title.
2. List all the authors.
3. List all author affiliations.
4. Insert the publication year.
5. Insert the article DOI.
6. In which country was the study conducted? (select all that apply)

- Australia
- Canada
- China

*(Additional countries listed with an option for the reviewer to add new countries if necessary)*

INTRODUCTION

1. How do authors frame the value of cultured meat? (select all that apply)
   - Promote animal welfares
   - Have the same sensory attribute. Experience as conventional meat
   - Reduce environmental pollution (GHG, water pollution, etc.)
   - Minimize the use of natural resources
   - Food security
   - Fulfil increasing demand for animal proteins
   - Food source for outer space expedition
   - Safer to consume than conventional meat
   - Healthier/ more nutritious than conventional meat
   - To replace conventional meat (with context)
   - To replace conventional meat (without context)

*(An option for the reviewer to add a new value(s) was provided)*

1. If you choose “to replace conventional meat (with context)”, quote the representative statement and include the page number.
2. If you choose “to replace conventional meat (without context)”, quote the representative statement and include the page number.

*(A space for the reviewer to include additional notes related to this section was provided)*

METHODS

1. How are the study participants selected? (select one that apply)

- Random
- Stratified
- Snowball

*(An option for the reviewer to add a new sampling method as seen necessary was provided)*

1. What are the survey methods used? (select all that apply)

- Online Questionnaire
- Paper Questionnaire
- Interview
- Conduct sensory evaluation

*(An option for the reviewer to add a new method as seen necessary was provided)*

1. What is the type(s) of sample used in the study? (select all that apply)

- Cultured meat/ meat product
- Image
- Conventional meat/ meat product
- Written information about cultured meat

*(An option for the reviewer to add a new type of sample as seen necessary was provided)*

1. What type(s) of meat/ meat product evaluated in this study? (select all that apply)

- Poultry
- Red meat (beef, pork, game)
- Seafood (fish, shrimp, etc.)
- Red meat products (burgers, meatballs, etc.)

*(An option for the reviewer to add a new type of meat as seen necessary was provided)*

1. What type(s) of cultured meat is evaluated? (select all that apply)

- Beef
- Chicken
- Fish

*(Additional types of meat listed with an option for the reviewer to add new types of meat if necessary)*

1. What product sensory attribute(s) is evaluated? (select all that apply)

- Appearance
- Texture
- Aroma
- Taste/ flavor

1. Is sensory attributes something that authors search for or an afterthought? (select one that apply)

- Primary outcome
- Secondary outcome
- Inference

1. How are opinions about sensory quality of CM elicited? (select one that apply)

- Subject asked about the taste of cultured meat
- Participants volunteer about hypothesis of cultured meat's taste
- Author makes hypothesis based on current technology

*(An option for the reviewer to add a new category as seen necessary was provided)*

*(A space for the reviewer to include additional notes related to this section was provided)*

RESULTS/ EVALUATIONS

1. What are the factors hypothesized for the acceptance of cultured meat? (select all that apply)

- Economic
- Psychology
- Animal welfare

*(Additional factors listed with an option for the reviewer to add new factors if necessary)*

1. What are the acceptance measures used to indicate cultured meat acceptance? (select all that apply)

- Willingness to try/ eat
- Willingness to buy/ pay
- Willingness to reduce meat consumption (may not be with a cultured meat)
- Willingness to replace conventional meat

*(An option for the reviewer to add a new measure as seen necessary was provided)*

1. What are the sensory measures used in the study? (select all that apply)***^a^***
   - Overall liking
   - Descriptive intensity rating
   - Discrimination testing (e.g. Triangle test)
   - Specific attribute liking
2. If you choose “specific attribute liking”, list the measured sensory attributes.***^a^***

CONCLUSION

1. What is the overall conclusion or key findings of this study?

ACKNOWLEDGEMENTS

1. List the study funding sources.
2. List the sources (providers) of the samples used in this study.
3. Where are study participants from (geographic location, institution, organization, etc.)
4. Quote the conflict of interest statement.

***^a^****By a collective agreement between authors (KVT and JL), data obtained from this question were removed due to insufficient information retrieval.*

# Appendix 3: Value framing of cell-cultured meat by authors of each study with all the extracted answers.

| **Value framing** | **Number of studies** | **Other terms/ answers included** | **References** |
| --- | --- | --- | --- |
| Food security | 7 |  | (Baybars et al., 2023; Lee et al., 2022; Leung et al., 2023; Lupton & Turner, 2018; Takeda et al., 2023; Verbeke et al., 2015a; Verbeke et al., 2015b) |
| Fulfil increasing demand of animal proteins | 6 |  | (Krings et al., 2022; Lee et al., 2022; Lupton & Turner, 2018; Mancini & Antonioli, 2020; Rolland et al., 2020; Verbeke, et al., 2015b) |
| As/ more nutritious than conventional meat | 8 | Healthier/ more nutritious than conventional meat; Have the same nutritional value as conventional meat | (Hallman & Hallman II, 2021; Krings et al., 2022; Ong et al., 2021; Possidónio et al., 2021; Rolland et al., 2020; Ruzgys & Pickering, 2020; Verbeke et al., 2015b; Vural et al., 2023) |
| Safer to consume than conventional meat | 11 | Health concerns of traditional meat consumption; reduce risk of zoonotic illnesses; causing new infectious disease | (Franceković et al., 2021; Krings et al., 2022; Leung et al., 2023; Pasitka et al., 2022; Possidónio et al., 2021; Rolland et al., 2020; Ruzgys & Pickering, 2020; Ryynänen & Toivanen, 2022; Verbeke, et al., 2015a; Verbeke et al., 2015b; Vural et al., 2023) |
| Minimize the use of natural resources | 18 | Sustainability | (Cornelissen & Piqueras-Fiszman, 2022; Grasso et al., 2019; Hallman & Hallman II, 2021; Lee et al., 2022; Leung et al., 2023; Lupton & Turner, 2018; Mancini & Antonioli, 2020; Pasitka et al., 2022; Possidónio et al., 2021; Rolland et al., 2020; Ruzgys & Pickering, 2020; Ryynänen & Toivanen, 2022; Slade, 2018; Takeda et al., 2023; Tucker, 2014; Verbeke et al., 2015b; Vural et al., 2023; Wilks & Phillips, 2017) |
| Reduce environmental pollution | 22 |  | (Baybars et al., 2023; Franceković et al., 2021; Grasso et al., 2019; Krings et al., 2022; Lee et al., 2022; Leung et al., 2023; Lunden et al., 2020; Lupton & Turner, 2018; Mancini & Antonioli, 2020; Ong et al., 2021; Pasitka et al., 2022; Possidónio et al., 2021; Rolland et al., 2020; Ruzgys & Pickering, 2020; Ryynänen & Toivanen, 2022; Slade, 2018; Takeda et al., 2023; Tucker, 2014; Verbeke et al., 2015a; Verbeke et al., 2015b; Vural et al., 2023; Wilks & Phillips, 2017) |
| Have the same sensory attributes/ experience as conventional meat | 6 |  | (Baybars et al., 2023; Hallman & Hallman II, 2020, 2021; Ong et al., 2021; Rolland et al., 2020; Slade, 2018) |
| Potential biodiversity loss | 2 | Biodiversity loss; endangering wildlife | (Grasso et al., 2019; Leung et al., 2023) |
| Reduce meat consumption | 1 |  | (Tucker, 2014) |
| Replace conventional meat | 6 |  | (Franceković et al., 2021; Hallman & Hallman II, 2020; Krings et al., 2022; Ruzgys & Pickering, 2020; Verbeke et al., 2015a; Wilks & Phillips, 2017) |
| Food source for outer space expeditions | 1 |  | (Benjaminson et al., 2002) |
| Promote animal welfares | 19 | More sustainable production; moral conflict (the meat paradox) | (Baybars et al., 2023; Cornelissen & Piqueras-Fiszman, 2022, 2022; Franceković et al., 2021; Hallman & Hallman II, 2020, 2021; Krings et al., 2022; Lee et al., 2022; Lupton & Turner, 2018; Mancini & Antonioli, 2020; Rolland et al., 2020; Ruzgys & Pickering, 2020; Ryynänen & Toivanen, 2022; Takeda et al., 2023; Tucker, 2014; Verbeke et al., 2015a; Verbeke et al., 2015b; Vural et al., 2023; Wilks & Phillips, 2017) |
| Less familiar to the public | 1 |  | (Lunden et al., 2020) |

# Appendix 4: The hypothesized determining factors of consumer acceptance of cell-cultured meat suggested by authors of each study.

| **Factors** | **Number of studies** | **Other terms/ answers included** | **References** |
| --- | --- | --- | --- |
| Animal welfare | 11 |  | (Baybars et al., 2023; Cornelissen & Piqueras-Fiszman, 2022; Franceković et al., 2021; Leung et al., 2023; Mancini & Antonioli, 2020; Ruzgys & Pickering, 2020; Ryynänen & Toivanen, 2022; Slade, 2018; Tucker, 2014; Verbeke et al., 2015b; Wilks & Phillips, 2017) |
| Environmental impact | 13 | Sustainability | (Baybars et al., 2023; Cornelissen & Piqueras-Fiszman, 2022; Franceković et al., 2021; Grasso et al., 2019; Leung et al., 2023; Lunden et al., 2020; Mancini & Antonioli, 2020; Possidónio et al., 2021; Ryynänen & Toivanen, 2022; Slade, 2018; Verbeke et al., 2015a; Verbeke et al., 2015b; Wilks & Phillips, 2017) |
| Framing/ labeling | 8 |  | (Cornelissen & Piqueras-Fiszman, 2022; Hallman & Hallman II, 2020, 2021; Krings et al., 2022; Mancini & Antonioli, 2020; Possidónio et al., 2021; Rolland et al., 2020; Ruzgys & Pickering, 2020) |
| Health/ nutrition/ safety | 18 | Need for government regulation | (Baybars et al., 2023; Franceković et al., 2021; Hallman & Hallman II, 2021; Krings et al., 2022; Leung et al., 2023; Lunden et al., 2020; Lupton & Turner, 2018; Mancini & Antonioli, 2020; Pasitka et al., 2022; Possidónio et al., 2021; Rolland et al., 2020; Ruzgys & Pickering, 2020; Ryynänen & Toivanen, 2022; Slade, 2018; Takeda et al., 2023; Verbeke et al., 2015a; Vural et al., 2023; Wilks & Phillips, 2017) |
| Psychology (food neophobia, disgust, belief in technology/ attitude) | 10 | Credible; attitude; ethichality; social norm | (Baybars et al., 2023; Cornelissen & Piqueras-Fiszman, 2022; Grasso et al., 2019; Leung et al., 2023; Lunden et al., 2020; Rolland et al., 2020; Ruzgys & Pickering, 2020; Takeda et al., 2023; Verbeke et al., 2015a; Vural et al., 2023) |
| Consumer knowledge about cultured meat | 11 | Familiarity; trendy/ interesting (curiosity) | (Cornelissen & Piqueras-Fiszman, 2022; Franceković et al., 2021; Grasso et al., 2019; Hallman & Hallman II, 2021; Lunden et al., 2020; Lupton & Turner, 2018; Possidónio et al., 2021; Rolland et al., 2020; Ruzgys & Pickering, 2020; Tucker, 2014; Wilks & Phillips, 2017) |
| Media coverage (including social media) | 1 |  | (Ryynänen & Toivanen, 2022) |
| Economic (price, income) | 10 |  | (Baybars et al., 2023; Franceković et al., 2021; Grasso et al., 2019; Lupton & Turner, 2018; Ruzgys & Pickering, 2020; Slade, 2018; Tucker, 2014; Verbeke et al., 2015a; Verbeke et al., 2015b; Wilks & Phillips, 2017) |
| Taste (tasty, have the same sensory experience as conventional meat) | 18 |  | (Baybars et al., 2023; Cornelissen & Piqueras-Fiszman, 2022; Franceković et al., 2021; Grasso et al., 2019; Hallman & Hallman II, 2021; Lee et al., 2022; Leung et al., 2023; Lupton & Turner, 2018; Mancini & Antonioli, 2020; Pasitka et al., 2022; Possidónio et al., 2021; Ruzgys & Pickering, 2020; Ryynänen & Toivanen, 2022; Takeda et al., 2023; Tucker, 2014; Verbeke et al., 2015a; Verbeke et al., 2015b; Vural et al., 2023) |
| Naturalness | 6 |  | (Baybars et al., 2023; Cornelissen & Piqueras-Fiszman, 2022; Lupton & Turner, 2018; Takeda et al., 2023; Verbeke et al., 2015a; Verbeke et al., 2015b) |
| Demographic background | 5 | age, diet, political affiliations, rural/ urban living, education | (Krings et al., 2022; Ryynänen & Toivanen, 2022; Slade, 2018; Tucker, 2014; Wilks & Phillips, 2017) |
| Convenience | 3 |  | (Grasso et al., 2019; Lupton & Turner, 2018; Verbeke et al., 2015a) |

# Appendix 5: Detailed list of potential conflict of interest of each study based on authors affiliation, study funding sources, and the conflict-of-interest statement.

Appendix 5_COI.xlsx

# Appendix 6: All data extracted in the data extraction step.

Appendix 6_All extracted data.xlsx
